# Pulmonary rehabilitation after severe exacerbation of COPD: a nationwide population study

**DOI:** 10.1186/s12931-023-02393-7

**Published:** 2023-04-07

**Authors:** Marina Guecamburu, Anaëlle Coquelin, Amandine Rapin, Nelly Le Guen, Agnès Solomiac, Pauline Henrot, Marie Erbault, Sandrine Morin, Maéva Zysman

**Affiliations:** 1 Service des Maladies Respiratoires et des épreuves fonctionnelles respiratoires CHU Bordeaux, 33604 Pessac, France; 2 Haute Autorité de la Santé, 93210 La Plaine Saint-Denis, France; 3 Département de Médecine Physique et de Réadaptation, Centre Hospitalo-Universitaire de Reims, CHU Reims, Hôpital Sébastopol, 51092 Reims, France; 4 Faculté de Médecine, Université de Reims Champagne-Ardenne, VieFra, EA3797, 51097 Reims, France; 5 Centre de Recherche Cardio-Thoracique de Bordeaux, U1045, CIC 1401, Univ-Bordeaux, 33604 Pessac, France

**Keywords:** Acute exacerbation of COPD, Pulmonary rehabilitation, Medical density, Health inequalities

## Abstract

**Background:**

Acute exacerbations of chronic obstructive pulmonary disease (COPD) lead to a significant reduction in quality of life and an increased mortality risk. Current guidelines strongly recommend pulmonary rehabilitation (PR) after a severe exacerbation. Studies reporting referral for PR are scarce, with no report to date in Europe. Therefore, we assessed the proportion of French patients receiving PR after hospitalization for COPD exacerbation and factors associated with referral.

**Methods:**

This was a national retrospective study based on the French health insurance database. Patients hospitalized in 2017 with COPD exacerbation were identified from the exhaustive French medico-administrative database of hospitalizations. In France, referral to PR has required as a stay in a specialized PR center or unit accredited to provide multidisciplinary care (exercise training, education, etc.) and admission within 90 days after discharge was assessed. Multivariate logistic regression was used to assess the association between patients’ characteristics, comorbidities according to the Charlson index, treatment, and PR uptake.

**Results:**

Among 48,638 patients aged ≥ 40 years admitted for a COPD exacerbation, 4,182 (8.6%) received PR within 90 days after discharge. General practitioner’s (GP) density (number of GPs for the population at regional level) and PR center facilities (number of beds for the population at regional level) were significantly correlated with PR uptake (respectively r = 0.64 and r = 0.71). In multivariate analysis, variables independently associated with PR uptake were female gender (aOR 1.36 [1.28–1.45], p < 0.0001), age (p < 0.0001), comorbidities (p = 0.0013), use of non-invasive ventilation and/or oxygen therapy (aOR 1.52 [1.41–1.64], p < 0.0001) and administration of long-acting bronchodilators (p = 0.0038).

**Conclusion:**

This study using the French nationally exhaustive health insurance database shows that PR uptake after a severe COPD exacerbation is dramatically low and must become a high-priority management strategy.

**Supplementary Information:**

The online version contains supplementary material available at 10.1186/s12931-023-02393-7.

## Introduction

Exacerbations of chronic obstructive pulmonary disease (COPD) are defined as events characterized by dyspnea and/or cough and sputum that worsens over less than 14 days [1]. Such exacerbations worsen symptoms, obstruct airflow, impact quality of life and increase the mortality risk, particularly among patients requiring hospitalization, hence called as severe exacerbation [2, 3]. Pulmonary rehabilitation (PR) is a global management approach that includes not only exercise training but also education and behavioral changes. In France, most PR programs are usually carried out over 3 to 6 weeks in dedicated centers, even if ambulatory care is increasingly prescribed. Guidelines strongly recommend PR after hospitalization for a COPD exacerbation [4–6] for its beneficial effect on exercise capacity, health-related quality of life and reduced readmissions and mortality [7, 8]. However, PR referral and uptake rates remain dramatically low [9]. For example, the referral rate ranged from only 1.9% to 30% in carefully selected populations, with fewer than 10% of patients completing the program [10–12].

The present study is the first to focus on PR uptake in a French exhaustive nationwide insurance database, covering more than 99% of the whole French population. Barriers to referral and uptake are complex and multi-factorial [13]. However, several factors are known to be associated with a higher PR uptake, such as younger age, living closer to a PR facility, lower comorbidity scores [9] and practitioner delivering PR [14]. For instance, among primary care physicians, a survey found that although two-thirds reported having PR available for their patients, only 38% routinely referred their COPD patients for it [15]. One of the greatest barriers to PR referrals is a lack of knowledge of PR among providers [16]. Identifying and mitigating risk factors for underutilization of PR at a national and international level is therefore essential.

Our first objective was to assess the rate of patients receiving PR after a severe exacerbation of COPD in France using the exhaustive French medico-administrative hospitalizations database. We also investigated which clinical and sociological factors could contribute to PR. Finally, we correlated PR uptake to medical density defined by the number of general practitioners (GPs) and pulmonologists relative to the same regional population in France. Similar analyses were performed for the number and facilities of PR centers, defined by the number of PR centers and number of beds relative to the same region.

## Methods

### Data sources

This retrospective study was based on the French health insurance database (SNDS, "Système National Des Données de Santé") from the 1st January to the 31st December 2017. The SNDS covers more than 99% of the French population and comprises three existing databases: the French health insurance database which contains all primary care reimbursed; the French national hospital discharge database (PMSI, Programme de médicalisation des systèmes d’information), which includes hospital diagnoses and medical procedures performed during each stay in French public and private hospitals; and the national death registry. The SNDS database includes information on the presence of long-term diseases (ALD, affection de longue durée) and reimbursable drugs. According to the International Classification of Diseases 10th revision (ICD 10), diagnoses are coded as principal diagnosis (PD: condition requiring hospitalization), related diagnosis (RD) and secondary associated diagnosis (SAD: complications and co-morbidities potentially affecting the course or cost of hospitalization) [17].

### Study population

**Fig. 1:**
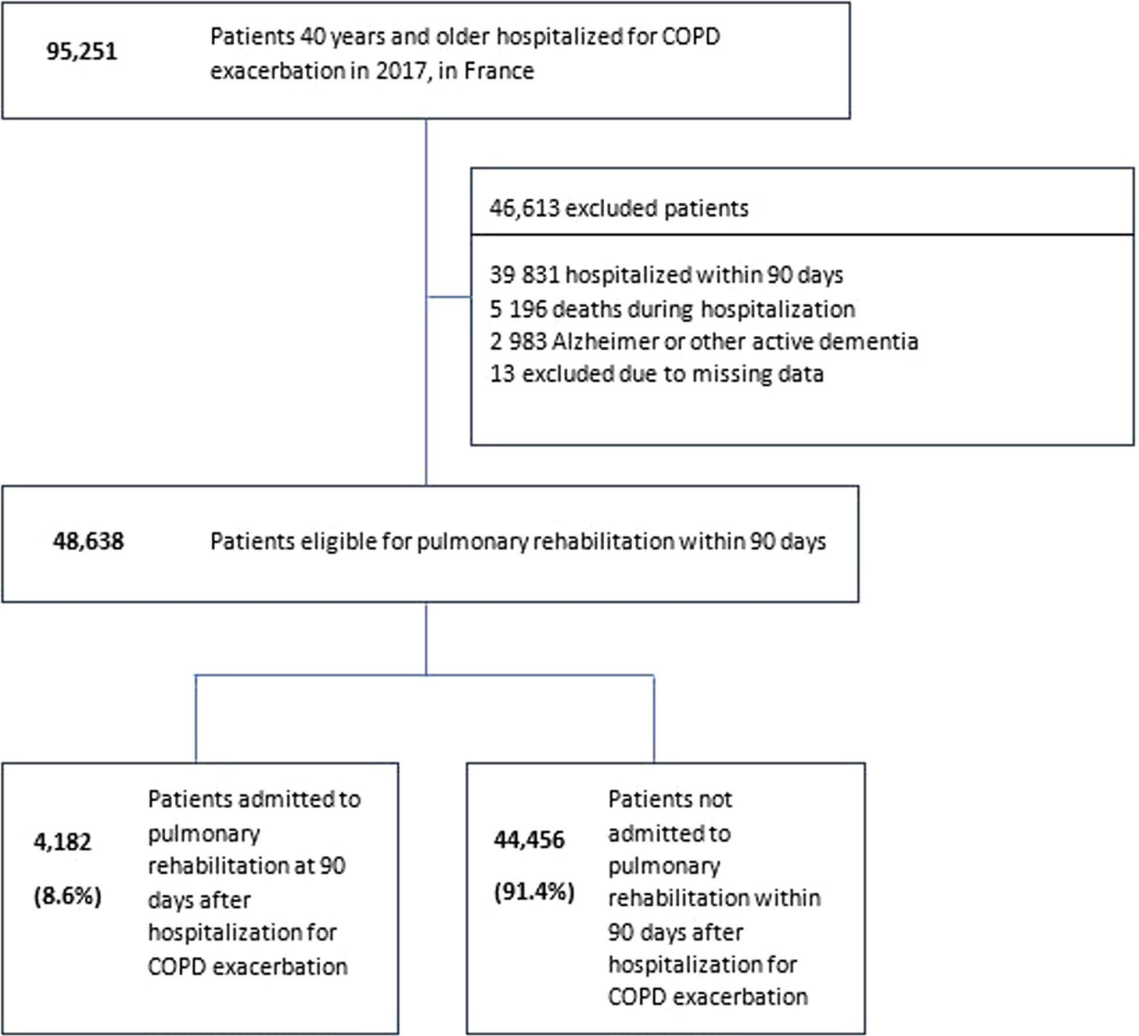
Flow chart

Our study included patients aged 40 years and older with a permanent address in France. Hospitalization was defined as an in-hospital stay of at least one night, ending in 2017, with a PD of COPD, or with a PD of COPD exacerbation associated with an SAD of COPD, or with a PD of a disease that may trigger a COPD exacerbation (influenza, pulmonary embolism, acute heart failure, acute respiratory failure, pneumothorax) associated with an SAD of COPD [18]. To focus on individuals who were systematically eligible for PR after a COPD exacerbation, we applied the following exclusion criteria, which were not mutually exclusive: (i) patients who died during stay, (ii) patients hospitalized for more than one night within 90 days after discharge or transfer to another acute care facility, hospice, long-term care facility, (iii) patients suffering from Alzheimer or other active dementia (Fig. 1).

### Outcome

PR was defined as a stay in a medical unit or a center entirely specialized in the management of respiratory diseases within 90 days of hospital discharge. These centers and units provide multidisciplinary care (exercise training, education, etc.) as recommended in international guidelines [1] and must meet a set of specifications in order to be coded as “PR center” or “unit” in the SNDS database. For patients with several stays, only the first was analyzed.

### Patients: spatial health inequalities and healthcare system

The Charlson comorbidity index (CCI) was used to estimate the weight of comorbidities the year before the study period [19, 20]. To facilitate the analyses, its principal components were gathered into major categories as described in [18]. In addition, the presence of a long-term chronic disease in 2017, called ALD (“Affection de Longue Durée”) in France, was also studied. The combined use of these two factors allowed us to distinguish between (a) comorbidities present at the time of admission to hospital, regardless of their severity (thanks to the CCI) and (b) comorbidities deemed serious enough to require an ALD.

Spatial health inequalities were estimated in terms of the French Deprivation Index (FDEP) and French Free Universal Health Care, (CMU-C, “Couverture Médicale Universelle Complémentaire”). The FDEP is based on four components (median household income, proportion of secondary school graduates among inhabitants aged 15 years and over, percentage of blue-collar workers in the active population, and proportion of unemployed) and 2013 is the most recent available data. The first quintile (Q1) corresponds to the 20% of the population living in the least deprived municipalities, while the fifth quintile (Q5) corresponds to the 20% living in the most deprived municipalities [21, 22].

Healthcare consumption in 2017 was described by the number of dispensations of inhaled long-acting bronchodilator (to estimate the background treatment), oxygen or non-invasive ventilation, contacts with healthcare providers (GPs and pulmonologists) and rehabilitation care in the year preceding the index hospitalization for COPD exacerbation. Analyses of the 7-day GP and 60-day pulmonologist follow-up excluded patients receiving PR respectively during the first 7 days and the first 60 days, because they were already receiving regular medical follow-up as part of their PR program and may not have had any additional external consultations.

### Ethics, consent and statistical analyses

This work was carried out by the HAS (Haute Autorité de Santé) whose aim is to design COPD-specific quality of care indicators based on French guidelines and workshops with experts (GPs, pulmonologists, expert patients, pharmacists, nutritionists, physiotherapists, physical and rehabilitation physician, professional pathologist, public health physician). According to French Law [19], this anonymous retrospective observational database study does not require approval by an ethics committee or informed signed consent from the patients.

Categorial variables were compared by Chi-square tests. Continuous variables were summarized as mean ± standard deviation or median [Q1–Q3] and were compared by Student tests, because of the sufficiently large sample size. The association between PR and hospitalization for COPD exacerbation was estimated by a multivariate logistic regression model and quantified by adjusted ORs (aOR) and their confidence intervals (95% CIs). Significance was set at p ≤ 0.05. The variables included in the multivariate model were chosen according to their clinical relevance. Analyses were performed using SAS Entreprise Guide version 7.15 software (SAS Institute Inc., Cary, NC, USA) and GraphPad Prism software.

## Results

### Patients’ characteristics

From the SNDS databases, we identified 95,251 patients aged ≥ 40 years with an in-hospital stay for a severe COPD exacerbation ending between the 1st January 2017 and the 31st December 2017 (Fig. 1).

At the time of index hospitalization, from the remaining eligible 48,638 patients admitted for a severe acute exacerbation of COPD, 66.2% had a PD of COPD. All others had COPD as an SAD and acute heart failure (14.3%), pneumonia (9.5%) or acute respiratory failure (5.9%) as PD. Subjects’ characteristics are described in Table 1. Briefly, median age was 75 [IQR 65–83] years, the majority were males (60.7%) and mortality rate at 6 months after hospital discharge was 7.8%. Regarding the FDEP, 50% of the patients were in the 4th and 5th quintiles corresponding to the most disadvantaged classes. The details of comorbidities assessed by ALD classification are shown in Additional file 1: Table S1 and concerned more than 80% of the patients. The median CCI was 5 [IQR 3–6], and the main comorbidities were diseases of the circulatory system, followed by diabetes mellitus, cancers and renal diseases, respectively corresponding to 32.4%, 22.3%, 15.2% and 7.1% of the patients.

**Table 1:** Characteristics of patients hospitalized for severe acute exacerbation in France, according to French national health insurance database (SNDS, “Système National De Santé”)

Characteristics of patients N = 48,638		
	N	%
Principal diagnosis		
COPD	32,177	66.2
Other principal diagnoses associated with secondary associated diagnosis of COPD		
Acute heart failure	6971	14.3
Pneumonia	4601	9.5
Acute respiratory failure	2876	5.9
Pulmonary embolism	855	1.8
Influenza	850	1.7
Pneumothorax	308	0.6
Gender		
Male	29,529	60.7
Age (years)		
Median [Q1–Q3]	75 [65–83]	
Charlson Index		
Median [Q1–Q3]	5 [3–6]	
Healthcare coverage		
French Free Universal Health Care / CMU	2521	5.2
French Deprivation Index*		
Q1	6390	13.5
Q2	7829	16.5
Q3	9635	20.4
Q4	10,827	22.9
Q5	12,651	26.7
At least one long-term disease (ALD) in 2017		
Presence	40,055	82.4
6-month mortality		
Death	3803	7.8
Long-acting bronchodilator prescription in 2017		
Median [Q1–Q3]	8 [1–11]	
Non-invasive Ventilation in 2017	1708	3.5
Oxygen therapy in 2017	6573	13.5

COPD: Chronic Obstructive Pulmonary Disease; SAD: secondary associated diagnosis; CMU: Couverture Médicale Universelle; SD: Standard Deviation

^*^French Deprivation Index: N = 1306 missing value (2.7%)

Regarding treatments, median long-acting bronchodilator prescription was 8 [IQR 1–11] per patient per year. However, 19.5% had no bronchodilator prescription and 66.5% of the patients had four drug deliveries or more. Finally, 13.5% of the patients received home oxygen therapy and 3.5% were treated with non-invasive ventilation. Furthermore, 38% of patients had a medical follow-up at day 7 and 28% a respiratory follow-up at day 60 (Additional file 1: Table S2).

### Main outcome

Among 48,638 patients admitted for a severe acute exacerbation of COPD, only 4,182 patients (8.6%, 7.6% among men versus 10.2% among women, p < 0.0001) received PR in the 90 days following discharge (Fig. 1). Moreover, COPD as PD was associated with a higher PR uptake (9.1% among patients with COPD as PD versus 7.6% among patients with COPD as SAD, p < 0.0001). The mean delay between discharge and the first rehabilitation care was 6.3 ± 17.6 days (Additional file 1: Table S3).

### Factors associated with PR

In univariate analysis (Table 2), patients who received PR were significantly, older (75.5 ± 11.6 versus 73.3 ± 12.2 years, p < 0.0001) and the proportion of women was higher (46.4% versus 38.6%, p < 0.00001). Considering ALD status, comorbidities were more frequent among patients receiving PR (86.5% versus 82.0%, p < 0.0001). According to the CCI, patients admitted to PR had significantly more chronic lung diseases (85.6% versus 76.8% p < 0.0001) the year before hospitalization. They had a lower socioeconomic status and a lower PR admission rate, according to the deprivation index and to data on beneficiaries of CMU-C. Compared to those without PR, a higher proportion of patients admitted to PR already received PR or outpatient physiotherapy sessions care during the year before the index COPD admission (46.3% versus 29.1%, p < 0.0001).

**Table 2:** Comparison of patients in France undergoing pulmonary rehabilitation or not after severe acute exacerbation, according to French national health insurance database (SNDS, “Système National De Santé”)

	No pulmonary rehabilitation		Pulmonary rehabilitation		p-value
	N (%)	%	N (%)	%	
	44,456	914	4182	8.6	
Gender					
Male	27,286	61.4	2243	53.6	< 0.0001
Age (years)					
Mean (± SD)	73.3 ± 12.2		75.5 ± 11.6		< 0.0001
6 months mortality					
Death	3278	7.4	525	12.6	< 0.0001
Healthcare coverage					
French Free Universal Health Care/CMU	2370	5.3	151	3.6	< 0.0001
French Deprivation Index					
Q1	5757	13.3	633	15.4	< 0.0001
Q2	7106	16.4	723	17.6	
Q3	8744	20.2	891	21.7	
Q4	9886	22.9	941	22.9	
Q5	11,724	27.1	927	22.5	
Non-invasive ventilation in 2017	1475	3.3	233	5.6	< 0.0001
Oxygen therapy in 2017	5812	13.1	761	18.2	< 0.0001
Rehabilitation care in year preceding hospitalization					
Yes	12,937	29.1	1937	46.3	< 0.0001
Long-acting bronchodilator prescription in 2017					
0	8726	19.6	749	17.9	0.0471
1	2685	6.0	238	5.7	
2	1875	4.2	174	4.2	
3	1690	3.8	154	3.7	
≥ 4	29,480	66.3	2867	68.6	
At least one long-term disease (ALD) in 2017					
Presence	36,439	82.0	3616	86.5	< 0.0001
Type of ALD					
Cardiovascular disease	19,594	44.1	1738	41.6	0.002
Respiratory disease	16,646	37.4	2207	52.8	< 0.0001
Metabolic disease	8690	19.6	649	15.5	< 0.0001
Tumor	6901	15.5	667	16.0	0.48
Charlson Index—5 principal components					
Chronic lung disease	34,121	76.8	3581	85.6	< 0.0001
Disease of circulatory system	14,388	32.4	1385	33.1	0.32
Diabete	10,057	22.6	785	18.8	< 0.0001
Cancer	6755	15.2	657	15.7	0.38
Renal disease	3228	7.3	233	5.6	< 0.0001

CMU: Couverture Médicale Universelle, ALD: affection de longue durée, COPD: Chronic Obstructive Pulmonary Disease, SD: Standard Deviation

Patients who received PR more often had at least four drug deliveries in 2017 (68.6% versus 66.3% p = 0.04). They also more often received oxygen therapy (18.2% versus 13.1%, p < 0.0001) and non-invasive ventilation (5.6% versus 3.3%, p < 0.0001) in 2017. Patients admitted to PR were more frequently followed by a pulmonologist after hospitalization (43.3% versus 31.7%, p < 0.0001). Finally, the mortality rate 6 months after hospital discharge was higher in the PR subgroup (12.6% versus 7.4%, p < 0.0001).

In multivariable analysis (Table 3), patients who received PR were more often women (aOR = 1.4, [1.3–1.5], p < 0.0001) and aged 60 years and older (aOR vary to 1.5 to 1.9 by age group per 5 years, p < 0.0001). According to the CCI, patients with comorbidities had significantly higher PR uptake (CCI ≥ 5, aOR = 2.9, [1.4–5.8], p < 0.0001). Healthcare consumption, defined as non-invasive ventilation and/or oxygen therapy and/or prescription of inhaled long-acting bronchodilators, was also significantly associated with PR (respectively aOR = 1.5, [1.4–1.6], p < 0.0001 and aOR 1.1 to 1.2, according to the number of deliveries ranging from 1 to more than 4, p = 0.0038).

**Table 3:** Multivariate analysis model by logistic regression of association between patient characteristics and pulmonary rehabilitation in France, according to French national health insurance database (SNDS, “Système National De Santé”)

	Multivariate analysis	
	aOR (95% CI)	p-value
Sex		
Male	1	
Female	1.36 (1.28–1.45)	< 0.0001
Age (years)		
< 50	1	
50–54	1.21 (0.86–1.70)	< 0.0001
55–59	1.41 (1.03–1.93)	
60–64	1.52 (1.10–2.09)	
65–69	1.47 (1.07–2.02)	
70–74	1.52 (1.10–2.10)	
75–79	1.37 (0.99–1.90)	
80–84	1.61 (1.16–2.23)	
≥ 85	1.94 (1.40–2.69)	
Charlson index		
0	1	
1–2	2.21 (1.11–4.41)	0.0013
3–4	2.82 (1.40–5.68)	
≥ 5	2.87 (1.42–5.80)	
Non-invasive ventilation and/or oxygen therapy in 2017		
No	1	
Yes	1.52 (1.41–1.64)	< 0.0001
Long-acting bronchodilator prescription in 2017		
0	1	
1	1.13 (0.97–1.32)	0.0038
2	1.18 (0.99–1.40)	
3	1.17 (0.97–1.40)	
≥ 4	1.19 (1.09–1.30)	

95% CI: 95% confidence interval; aOR: OR adjusted

### Geographic analyses of PR disparities

**Fig. 2:**
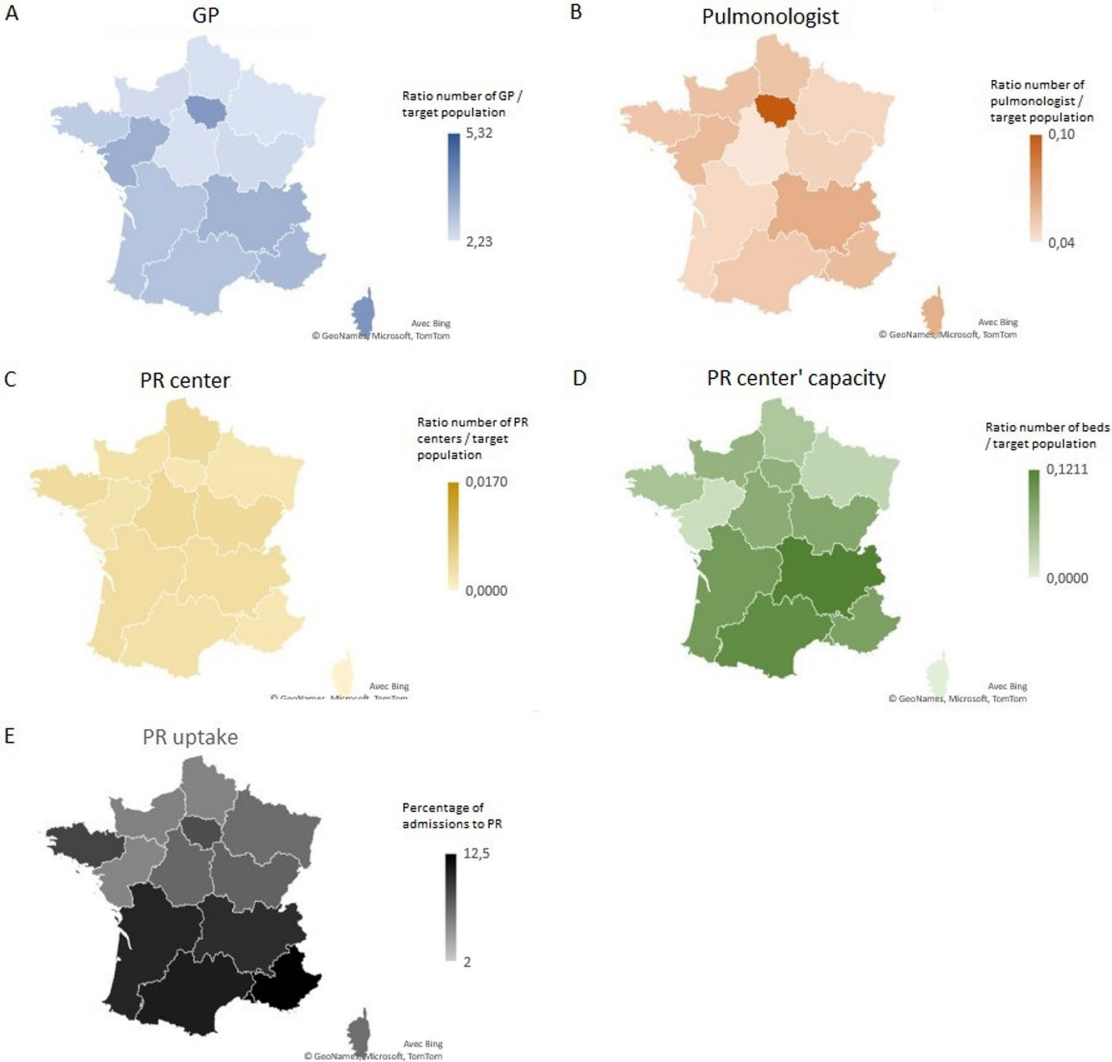
Healthcare resources per region (**A** GP, **B** pulmonologist, **C** PR centers, **D** Number of beds in PR centers). **E** PR uptake assessed by admission rate to PR in each region. GP: general practitioners, PR: pulmonary rehabilitation

**Fig. 3:**
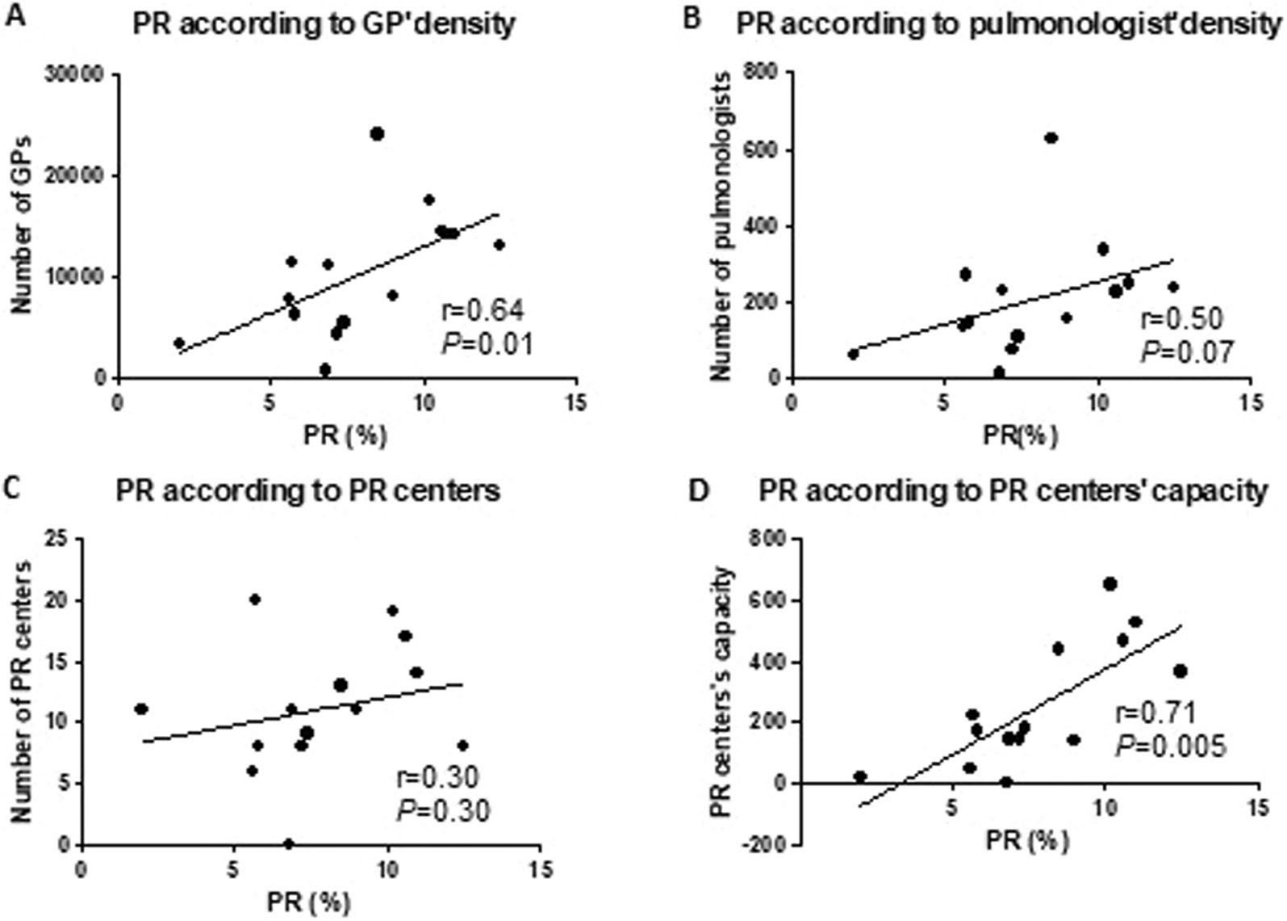
Pulmonary rehabilitation uptake according to number of physicians. **A** GP density, **B** pulmonologist density, **C** pulmonary rehabilitation centers, **D** pulmonary rehabilitation center capacity (based on number of beds), from the SIRSé (Système d’Information Interrégional en Santé). GPs: general practitioners, PR: pulmonary rehabilitation

Geographical variations concerning PR uptake were also evaluated (Fig. 2). As expected, we noted regional disparities, from 2% in the overseas departments to 12.5% in southeast France (Provence-Alpes-Cote d'Azur Region). Interestingly, GP density was significantly correlated with PR uptake, whereas pulmonologist density was not (respectively r = 0.64, p = 0.01 and r = 0.50, p = 0.07, Fig. 3A, B). PR center facilities, according to the number of beds in conventional hospitalization and the number of ambulatory places, were significantly correlated with PR uptake (r = 0.71, p = 0.005, Fig. 3D), but not with PR center number.

## Discussion

In this large population database covering more than 99% of the entire French population, we found that among the 48,638 eligible patients, only 4,182 (8.6%) received PR within the 90 days after discharge for a severe exacerbation of COPD in 2017, a figure that is dramatically low in view of the recommendations [4–6]. COPD as PD was related to a higher PR uptake because respiratory symptoms may be at the forefront for these patients. In the USA, Spitzer et al. [12] found 1.9% of PR uptake 6 months after hospitalization for a COPD exacerbation and 2.7% at 12 months. Vercammen-Grandjean et al*.* [23] reported similar findings. Both highlight the gap between recommendation and practice. Our study is the first to report such a result in a European country. Although very low, our rate seems higher, especially since we chose a 3-month delay, whereas Spitzer et al. [12] chose 6 months and Vercammen-Grandjean et al. [23] 12 months. The design of the French health care system, where PR programs after a severe exacerbation of COPD are usually carried out in subacute care in-patient post-hospitalization over 3 to 6 weeks could have contributed to low PR uptake [24].

No formal validation of the ideal interval between discharge and rehabilitation exists in the literature. Based on a systematic review including 13 randomized controlled trials [25], the GOLD report highlighted a reduction in hospitalization in patients who had an exacerbation within the previous 4 weeks [1]. Nevertheless, a large US cohort of more than 190,000 patients hospitalized for COPD reported a significant association between PR initiation within 90 days after discharge and a lower risk of mortality and fewer re-hospitalizations at one year [8, 9]. At the very least, research considering interventions to improve or maintain physical activity in the immediate post-discharge period following COPD exacerbation is needed to test whether this can improve outcomes and reduce the risk of readmission [26].

### Features related to PR uptake

Controversial results exist concerning gender. A higher proportion of women were admitted to PR in Vercammen-Grandjean et al. [23] as in the present study contrary to what was published by Spitzer et al. [12]. Compared to men, women with COPD have a lower quality of life, face a more rapid decline in lung function [27] and more frequent anxiety and depression, which could lead to higher healthcare consumption [28]. Moreover, Souto-Miranda et al*.* [29] showed that women suffer more activity-related dyspnea, severe hyperinflation, frequent exacerbation and hospitalization, which could explain why women were more frequently referred for PR.

We found that patients who were older, who required more oxygen or NIV or who had more comorbidities were significantly more likely to receive PR. They also received more often PR or outpatient physiotherapy sessions the year before the index COPD admission. Taken together, these results demonstrate that disease severity is an important factor associated with PR uptake. Regarding the age of patients, our results differ from those of other studies [12, 23] and highlight the impact of the organization of healthcare. Although the SNDS database does not contain precise clinical information, we think that some elderly and comorbid patients could correspond to Fried's frailty concept (weight loss, exhaustion, low physical activity, slowness and weakness) [30]. In a cohort of 816 COPD patients aged 70 ± 10 years, Maddock et al. [31] showed that 61.3% of patients admitted to rehabilitation no longer had frailty criteria after their stay, meaning that patients with more severe COPD may derive a greater benefit from PR. Also, older patients with more frequent co-morbidities or patients with a more severe COPD, assessed by requiring ventilatory support or oxygen therapy, have greater functional limitations and experience a negative impact on their activities of daily living, which may explain the better PR uptake. However, PR is indicated much earlier in the management of COPD patients and not only at this level of severity, which is a major point for improvement**.**

As found elsewhere, lower socioeconomic status, according to the CMU-C and the deprivation index, seems to be associated with lower PR uptake and could also be a target for improvement [32, 33].

### Healthcare system and PR

The distance between home and rehabilitation center are well established factors of failure [34]. PR uptake varied from 2 to 12.5% according to the different regions in our study. The study design did not allow us to discriminate the rates of PR participation in accordance to living area of each patient, however we have shown that PR uptake is correlated to PR facilities in the same region highlighting the importance of helping easy access. Consistent with this findings, Hug et al*.* [35] reported that environmental context, resource factors (travel distance, transport, parking, difficulties in fitting programs in with work or family obligations) were frequent barriers for PR uptake.

We also found a correlation between regional GP density and PR uptake. In a study of 252 primary care professionals in UK, Watson et al. [36] showed that those who had a respiratory qualification (63%) referred more patients to rehabilitation (59.1%) than those without it (32.2%), again emphasizing the crucial role of GPs in patient management.

We noticed that 19.5% of the patient did not have any bronchodilator prescription during the study period. It may be explained by a lack of therapeutic education or adherence to treatment however, we can not exclude a lack of prescription due to the design of our study. Various possibilities for improvement exist such as educational programs, electronic tools, collaboration with GPs and providers. One of the options would be to promote flexibility in PR programs regarding schedules and location. The development of tele-rehabilitation techniques could allow better adherence and better access for the most isolated or working patients [37], especially since this is a safe practice and with results possibly close to those of traditional center-based pulmonary rehabilitation [38, 39]. Social isolation and lack of intrinsic motivation are also barriers to rehabilitation [35]. Patient associations could be a major contributor in promoting the benefits of PR by giving direct feedback to the most isolated or anxious patients.

Our work has several strengths that distinguish it from previous publications thanks to the exhaustiveness of the French national healthcare insurance database. It is the first to focus on PR uptake in the entire French insurance database with such a high number of patients. However, the study also has some limitations. First, undiagnosed COPD patients were not included in the analysis, yet one of the major issues of this disease is under-diagnosis [40]. Nevertheless, severe acute COPD of requiring hospitalizations concerns rather the advanced stages of COPD, where hopefully diagnosis is available relatively more often. Second, the potential for unmeasured confounding remains. The SNDS database allows the accurate identification of certain factors, including readmissions, death, comorbidities, and reginal characteristics. However, it lacks significant additional details such as objective data for disease severity (Pulmonary Function Testing), adherence to recommended pharmacotherapy, willingness to participate in PR program, details about number and type of PR sessions delivered, and other social, psychological and environmental factors that are documented barriers to initiating PR. Among people with COPD who are suitable for PR, fewer than a half were really referred to PR programs, yet we are unable to determine whether physicians failed to refer patients for PR or whether the latter chose not to enroll [35]. Finally, we focused on in-patient PR, even though ambulatory strategies are emerging and should be considered in the future to confirm our data in this population.

Despite these limitations, the current study underscores some critical issues that deserve attention. PR uptake after a severe exacerbation of COPD is unacceptably low and is very heterogeneous around in France. Geographic inaccessibility for many deserving patients and subsequent health disparities remain a major issue [12]. While we focused on PR in health care institutions, we now need data on outpatient PR through specific coding and its promotion by the French health insurance system.

## Conclusion

In the French national health insurance database, PR after a severe COPD exacerbation was received by less than a tenth of the COPD population and was associated with the density of medical practitioners and PR center facilities. To move personalized COPD medicine forward, we identified several patient-related (age, gender, comorbidities) and social risk factors (lower socio-economic status) associated with an increased risk of non-uptake of PR. Strategies to promote PR and to reinforce strong collaboration between healthcare establishments and primary care as well as pulmonologists and GPs could increase PR uptake after a severe COPD exacerbation. Home-based programs and tele-rehabilitation could become one of the key solutions to promote greater availability and accessibility to PR.

## Supplementary Information

**Additional file 1: Table S1.** Characteristics of patients with detailed Charlson components, long-term disease, and treatment prescription. **Table S2.** Medical follow-up after discharge. **Table S3.** Time to rehabilitation care uptake after index severe exacerbation of COPD.

## Data Availability

The datasets used and/or analyzed in the study are available from the corresponding author on reasonable request.

## Abbreviations

ALD: Affection longue durée (presence of long-term disease)
CMU-c: Couverture médicale universelle complémentaire (French free universal health care)
COPD: Chronic Obstructive Pulmonary Disease
FDEP: French deprivation index
GP: General practitioners
HAS: Haute Autorité de Santé (health authority whose objective is to design specific COPD quality of care indicators)
ICD 10: International classification of disease 10th revision
PD: Principal diagnosis
PMSI: Programme de médicalisation des systems d’information (French national hospital discharge database which includes hospital diagnosis and medical procedures performed during each stay in French public and private hospitals
PR: Pulmonary rehabilitation
RD: related diagnosis
SAD: secondary associated diagnosis
SNDS: Système National des Données de Santé (central French health insurance linking the other databases mentioned above such as PMSI and DCIR)
