# Supplementary material for: Pulmonary rehabilitation after severe exacerbation of COPD: a nationwide population study
Source: Respir Res. 2023 Apr 7;24:102. doi: 10.1186/s12931-023-02393-7 (PMC10082500; doi:10.1186/s12931-023-02393-7)
Supplement: Supplementary file 1 — Additional file 1: Table S1. Characteristics of patients with detailed Charlson components, long-term disease, and treatment prescription. Table S2. Medical follow-up after discharge. Table S3. Time to rehabilitation care uptake after index severe exacerbation of COPD. [file 12931_2023_2393_MOESM1_ESM.docx]

**Table S1:** Characteristics of patients with detailed Charlson components, long-term disease, and treatment delivery

| **Characteristics of patients  N = 48,638** | | |
| --- | --- | --- |
|  | **N (%)** | **%** |
| **Charlson Index** | | |
| mean (+/- SD) | 4.8 +/- 2.4 | |
| **Charlson Index - 5 principal components** |  |  |
| Chronic lung disease | 37,702 | 77.5 |
| Disease of circulatory system | 15,773 | 32.4 |
| Diabetes | 10,842 | 22.3 |
| Cancer | 7,412 | 15.2 |
| Renal disease | 3,461 | 7.1 |
| **At least one long-term disease (ALD) in 2017** | | |
| Presence | 40,055 | 82.4 |
| **Type of ALD** |  |  |
| Cardiovascular disease | 21,332 | 43.9 |
| Respiratory disease | 18,853 | 38.8 |
| Metabolic disease | 9,339 | 19.2 |
| Tumor | 7,568 | 15.6 |
| Mental disease | 3,944 | 8.1 |
| **Long-acting bronchodilator delivery in 2017** |  |  |
| No delivery | 9,475 | 19.5 |
| 1 delivery | 2,923 | 6.0 |
| 2 deliveries | 2,049 | 4.2 |
| 3 deliveries | 1,844 | 3.8 |
| ≥ 4 deliveries | 32,347 | 66.5 |

ALD: affection de longue durée, SD: standard deviation

**Table S2:** Medical follow-up after discharge

| **Medical follow-up after discharge**  **N = 48,638** | | |
| --- | --- | --- |
|  | **N** | **%** |
| **GP follow-up at day** **7*** | 18,417 | 37.8 |
| **Pulmonologist follow-up at day 60**** | 13,573 | 27.8 |

GP : general practitioner

*: 11.0% of missing data (N=5 363), **: 12.4% of missing data (N=6 017)

**Table S3:** Time to rehabilitation care uptake after index severe exacerbation of COPD

| **Characteristics of patients with rehabilitation care N = 4,182 - 8.6%** | | |
| --- | --- | --- |
|  | **N (%)** | **%** |
| **Time from discharge to rehabilitation care (days)** | | |
| 0-7 | 3,582 | 85.65 |
| 8-14 | 100 | 2.4 |
| 15-30 | 138 | 3.3 |
| 31-60 | 196 | 4.7 |
| 61-90 | 166 | 4.0 |
| **Time from discharge to rehabilitation care** | | |
| mean (+/- SD) | 6.3 +/- 17.6 | |

SD: standard deviation
